# Intracellular Sphingosine‐1‐Phosphate Induces Lipolysis Through Direct Activation of Protein Kinase C Zeta

**DOI:** 10.1096/fj.202403272R

**Published:** 2025-04-07

**Authors:** Sarah Weske, Melissa Kim Nowak, Alex Zaufel, Lea Esser, Christoph Peter, Lisa Walz, Helena Kühn, Tsyon Wolde, Julia Hoppe, Nathalie Hannelore Schröder, Tobias Buschmann, Philipp Wollnitzke, Bodo Levkau

**Affiliations:** ^1^ Institute for Molecular Medicine III, Medical Faculty and University Hospital Düsseldorf Heinrich Heine University Düsseldorf Germany; ^2^ Department of Gastroenterology, Hepatology and Infectious Diseases, Medical Faculty and University Hospital Düsseldorf Heinrich Heine University Düsseldorf Germany; ^3^ Institute for Molecular Medicine I, Medical Faculty and University Hospital Düsseldorf Heinrich Heine University Düsseldorf Germany; ^4^ Cardiovascular Research Institute Düsseldorf (CARID) Düsseldorf Germany

**Keywords:** adipocytes, lipidomics, lipolysis, S1P lyase, S1P receptors, sphingosine‐1‐phosphate (S1P)

## Abstract

Dysregulated sphingosine‐1‐phosphate (S1P) signaling has been associated with obesity, insulin resistance, and type II diabetes. As metabolic disorders are intricately interrelated, studies on S1P effects explicitly on lipolysis have been scarce, particularly as S1P has also effects on adipogenesis, with studies implicating extracellular and intracellular mechanisms. Here, we have concentrated on the latter, as 10–50 μM S1P potently increased lipolysis in differentiated 3T3‐L1 adipocytes, whereas S1P concentrations sufficient to activate S1P receptors (S1PRs; 0.1–1 μM) or S1PR agonists had no effect. Neither was ceramide increased by S1P, nor was S1P‐mediated lipolysis affected by the ceramide synthase inhibitor Fumonisin B1. In contrast, inhibition of protein kinase C zeta (PKC zeta) completely abrogated S1P‐mediated lipolysis. S1P also induced Thr410 phosphorylation of PKC zeta in 3T3‐L1 adipocytes and activated recombinant PKC zeta in kinase assays. S1P‐mediated lipolysis was dependent on hormone‐sensitive lipase (HSL) and relied mechanistically on PKC zeta activation of MAPK to phosphorylate HSL at Ser660. Inhibition of S1P degradation by blocking the S1P lyase through VD‐78 also increased lipolysis in 3T3‐L1 cells and primary adipocytes. S1P lyase inhibition by 4‐Deoxypyridoxine (DOP) in mice rendered obese by a 10‐week high‐fat diet (HFD) for an additional 6 weeks, concomitantly with the HFD, reduced white gonadal adipose tissue (gWAT) mass and diminished adipocyte size in gWAT and inguinal WAT, and increased free fatty acid in plasma and gWAT. PKC zeta phosphorylation and activity, as well as HSL Ser660 phosphorylation, were increased in gWAT of DOP‐treated mice. This study assigns lipolysis as the first physiological function of PKC zeta activation by S1P and identifies an exclusive adipocyte‐specific aspect of S1P function in obesity.

## Graphical abstract

We have identified intracellular S1P as a direct activator of PKC zeta, leading to potent activation of lipolysis in 3T3‐L1 and primary adipocytes in vitro, while inhibition of S1P degradation by blocking S1P lyase through VD‐78 further enhances this effect. In vivo, inhibition of S1P lyase by 4‐Deoxypyridoxine (DOP) in HFD‐fed mice enhanced lipolytic activity, resulting in diminished adipose tissue mass and activation of PKC zeta and HSL in gWAT.

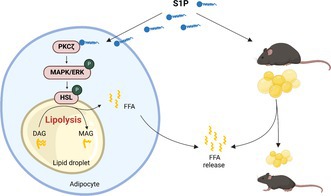

## 1 Introduction

Dysregulated S1P metabolism and signaling as well as altered plasma S1P concentrations have been associated with obesity, insulin resistance, type II diabetes, and metabolic dysfunction‐associated steatotic liver disease (MASLD) [1, 2, 3]. As such disorders are intricately interrelated, studies on the direct effects of S1P explicitly on lipolysis and/or adipogenesis have been scarce. Early studies have shown that the S1P generating enzyme sphingosine kinase 1 (SK1) was induced in mouse 3T3‐L1 pre‐adipocytes during differentiation and promoted adipogenesis [4]. Several studies by the Park group have demonstrated that exogenously added S1P inhibited 3T3‐L1 differentiation by downregulating adipogenic transcription factors and inactivating JNK and p38 MAPK signaling [5] and have implicated the S1P receptor 2 (S1PR2) as being responsible [6, 7]. However, the same authors have also shown that the functional S1PR antagonist FTY720 that engages all S1PRs except S1PR2 suppressed differentiation and induced lipolysis in 3T3‐L1 cells and inhibited adipogenesis and promoted lipolysis in mice on a high fat diet [8] suggesting other mechanisms. S1P also induced the transdifferentiation of orbital fibroblasts into adipocytes, a hallmark of Graves' orbitopathy, and S1PR1 was identified as the receptor involved [9].

We have previously demonstrated that S1P signaling through S1PR2 attenuated adipogenesis in mesenchymal precursor cells in favor of osteogenesis by suppressing PPARγ‐dependent gene transcription [10]. Pharmacological targeting of this mechanism using a S1PR2 agonist was then shown to be effective in treating mouse osteoporosis [11]. Studies on the phenotype of S1PR2‐deficient mice in respect to adipose tissue are controversial as Kitada and co‐workers have observed a leaner and more favorable metabolic phenotype and smaller adipocytes under a high fat diet [12], whereas we have described S1PR2^−/−^ mice as more obese than controls [10] although both agree on S1PR2 promoting adipogenic differentiation.

However, other studies have suggested S1PR‐independent effects by demonstrating that S1P inhibited adipogenic differentiation of mouse 3T3‐L1 and human SGBS pre‐adipocytes at concentrations higher than 1 μM [13]. Furthermore, S1P concentrations up to 30 μM have been shown to induce lipolysis in a cAMP‐dependent manner in differentiated rat white adipocytes [14]. Such S1P concentrations are far beyond the nanomolar Kds of S1PRs, suggesting other S1PR‐independent mechanisms. In support, Cowart's group has demonstrated that mice lacking SK1 in adipocytes gained more weight on a high‐fat diet and featured hypertrophied adipocytes with low lipolytic activity without any hint of S1PRs being involved [15]. Thus, both extracellular S1PR‐mediated signaling and intracellular S1P actions must be considered to explain the diverse and complex S1P effects on adipocyte biology. In this study, we have concentrated on the latter by exploring S1P actions occurring at concentrations beyond the range of S1PR signaling. We have identified S1P to induce lipolysis by directly activating PKC zeta in differentiated 3T3‐L1 adipocytes in vitro and in high‐fat diet‐induced obesity in mice in vivo.

## 2 Materials and Methods

### 2.1 Mouse Models

C57BL/6J mice were obtained from Charles River Laboratories. Mice were maintained at ambient temperature (22°C) under a 12 h:12 h light–dark cycle. Samples were obtained from male mice. All mice were fed a high fat diet (C1090‐60, Altromin) for 6–10 weeks. Then, one group of mice continued to be fed a high fat diet, while the other group was switched to a high fat diet +4‐deoxypyridoxine (DOP). DOP was administered via drinking water at 180 mg/L (30 mg per kg body weight per day) for 6 weeks. Mouse weights were measured every week. Plasma and tissue samples were taken after a 12 h overnight fast and stored immediately at −80°C. All procedures were approved by and in accordance with the institutional guidelines of the Landesamt für Natur, Umwelt und Verbraucherschutz NRW, Germany. Animal studies are reported in compliance with the ARRIVE guidelines [16]. A total of 40 mice were used in this whole study. Every effort was made to minimize the number of animals used and their suffering.

### 2.2 Cell Culture

3T3‐L1 preadipocytes were obtained from the European Collection of Cell Culture (86052701, ECACC, Salisbury, UK). Differentiation was performed according to the supplier's instructions using DMEM containing 10% w/v FBS with 0.25 μM dexamethasone, 0.5 mM 3‐Isobutyl‐1‐methylxanthine (IBMX), 2 μM Rosiglitazone, and 1 μg/mL insulin (Merck) for 3 days + 2 days of DMEM containing 10% w/v FBS with 1 μg/mL insulin (Merck). After that, cells were incubated in DMEM containing 10% w/v FBS until cells were fully differentiated after a total of 14 days. The medium was changed every 2 days.

### 2.3 Isolation and Differentiation of Murine Primary Adipocytes

Isolation of murine primary adipocytes from mouse gonadal white adipose tissue (gWAT) was carried out following a modified protocol from Oeckl et al. [17]. The tissue was dissected and rinsed with PBS before being transferred to a 15 mL tube containing 5 mL collagenase solution (HBSS + CaCl_2_ and MgCl_2_, 1% antibiotic‐antimycotic, 2.5% (w/v) BSA, 0.2% (w/v) collagenase type II (#C2‐22, Sigma‐Aldrich), pH 7.4). Digestion was performed for 1 h at 37°C, with vigorous shaking by hand every 10 min. The digested tissue was filtered through a 100 μM cell strainer and the tube was rinsed with an equal volume of wash buffer (HBSS, 3.5% (w/v) BSA, 1% antibiotic‐antimycotic), which was subsequently passed through the cell strainer. Cells were centrifuged at 250 × *g* for 5 min at room temperature. The tube was carefully inverted several times before a second centrifugation step was performed. Subsequently, the fat layer and supernatant were removed, and erythrocyte lysis was performed using 1 mL of erythrocyte lysis buffer (154 mM NH_4_Cl, 10 mM K_2_HPO_4_, 0.1 mM EDTA in ddH_2_O, pH 7.4) for 5 min at room temperature. The reaction was stopped by adding 10 mL of wash buffer and the cells were centrifuged at 500 × *g* for 5 min. Supernatant was removed, cells were resuspended in 10 mL of growth medium (DMEM 4.5 g/L glucose, L‐glutamine, 10% FCS, 1% antibiotic‐antimycotic) and the cell suspension was filtered through a 40 μM cell strainer before seeding into cell culture plates. Cells were allowed to adhere by incubation at 37°C and 5% CO_2_ overnight before changing the medium the next day. Growth medium was changed every other day until cells reached 80%–100% confluency. To induce differentiation, the medium was changed to double the volume of induction medium (growth medium +850 nM insulin, 1 μM rosiglitazone, 1 mM dexamethasone, 0.5 mM IBMX). On Day 3 of differentiation, the medium was changed to differentiation medium (growth medium +850 nM insulin), which was changed every other day until the cells were fully differentiated on Day 8.

### 2.4 Western Blot Analysis

Isolation of total protein of differentiated 3T3‐L1 cells after treatment with 10 μM S1P in 0.02% BSA (D‐erythro Sphingosine‐1‐Phosphate, Enzo) for 15 min with or without preincubation with 3 μM Bisindolylmaleimide I (Merck) for 1 h was performed in RIPA Buffer + Halt Protease‐ und Phosphatase‐Inhibitor‐Cocktail (Thermo Scientific), followed by a 30 min incubation on ice. Cell lysates were clarified via centrifugation at 14 000 × *g* at 4°C for 10 min and were stored at −80°C. Isolation of total protein from 40 mg gWAT of C57BL/6 J mice was performed by tissue disruption using a handheld rotor‐stator homogenizer (TissueRuptorII, Qiagen) in RIPA Buffer + Halt Protease‐ und Phosphatase‐Inhibitor‐Cocktail (Thermo Scientific) and followed with incubation on ice for 30 min. Protein concentrations were determined using a BCA protein assay (Thermo Scientific). A total of 30 μg protein was subjected to SDS‐PAGE, and proteins were then transferred to polyvinylidene difluoride (PVDF) membranes. Target protein detection was performed after blocking in 5% nonfat milk–PBS + Tween20 for 1 h before incubation with primary antibodies (phospho‐p44/42 MAPK (Erk1/2) (Thr202/Tyr204) no. 9101, p44/42 MAPK (Erk1/2) no. 9102, phospho‐HSL (Ser660) no. 45804, HSL no. 4107, Cell Signaling, p‐PKC zeta (H‐2) no. sc‐271962 and PKC zeta (H‐1) no. sc17781, Santa Cruz Biotechnology, Anti‐GAPDH no. 5G4cc, HyTest) at 4°C overnight. Membranes were washed 3 times in PBS with 0.5% milk and 0.05% Tween‐20 and exposed to secondary antibodies (peroxidase‐labeled anti–mouse IgG (H + L) no. PI‐2000 or anti‐rabbit IgG (H + L) no. PI‐1000, Vector). Signals were visualized using enhanced chemiluminescence solution (Immobilon Forte Western HRP Substrate no. WBLUF0100; Millipore) and were detected using ChemiDoc XRS + Imaging System (BioRad). All experiments are performed as biological replicates.

### 2.5 Lipolysis Assay

Glycerol release from differentiated 3T3‐L1 cells and primary adipocytes was measured 16 h after induction of lipolysis in the presence or absence of 10 μM S1P, 10 μM AUY954 (Cayman Chemical), 10 μM CYM5520 (Cayman Chemical), 10 μM CYM5541 (Cayman Chemical), 1 μM VD‐78 (Novartis) or 1 μM isoproterenol (Merck) with or without pre‐incubation with 3 μM Bisindolylmaleimide I, 10 μM PKC zeta pseudo‐substrate inhibitor no. sc‐3098 (Santa Cruz), 25 μM small molecular HSL inhibitor no. NNC0076‐0079 (Novo Nordisk), 10 μM PD98059 (SelleckChemicals), 100 nM Wortmannin (SelleckChemicals), 100 μM SQ22536 (SelleckChemicals) or 25 μM Fumonisin B1 (Cayman Chemical) for 1 h. After incubation, cell culture supernatants were collected. 80 μL of supernatants were incubated with 250 μL Hydrazine buffer (50 mM Glycine (pH 9.8), 0.05% Hydrazine Hydrate, 1 mM MgCl_2_, 0.75 mg/mL ATP, 0.375 mg/mL NAD, 25 μg/mL GDH, 0.5 μg/mL Glycerokinase, all Merck) for 2 h at RT. Absorbance (OD 340 nm) was measured using a microtiter plate reader (CLARIOstar Plus, BMG LabTech). Glycerol concentration was calculated by Glycerol standard curve. All experiments are performed as biological replicates.

### 2.6 Plasma Free Fatty Acids

Plasma free fatty acids were measured by Free Fatty Acid Assay Kit no. ab65341 (Abcam) according to the manufacturer's instructions.

### 2.7 Histological Analysis

For hematoxylin and eosin (H&E) staining, perigonadal WAT of mice was fixed in 4% paraformaldehyde (PFA) overnight at 4°C, followed by dehydration in 70% ethanol. After dehydration, tissues were embedded in paraffin, sectioned at a thickness of 6 μm, and stained with H&E following the standard protocol. Images of perigonadal WAT samples were captured using the Keyence BZ‐X Microscope (Keyence) and analyzed using *Fiji‐*Adiposoft (*Fiji Is Just ImageJ, version 2.9.0*). Adipocyte diameter and area were calculated from two different slides with a quantification of at least three fields of view at 100‐fold magnification.

### 2.8 S1P Stimulation for LCMS Measurements of Ceramides

Differentiated 3T3‐L1 cells were treated with 10 μM S1P (Avanti Research) for 30 min, washed with ice‐cold PBS, harvested with MeOH (300 μL) and spiked with 10 μL ISTD (see LCMS measurements). Samples were precipitated (−80°C, overnight), centrifuged (10 000 × *g*, 15 min) and the supernatant transferred into 300 μL glass vials. Samples were stored at (−80°C) prior to measurement. All experiments are performed as biological replicates.

### 2.9 FFA Derivatization for LCMS

Derivatization Solvents (LCMS grade) were distilled over a Vigreux column (Sigma) and WAT tissue was stored at −80°C prior to sample preparation. 1–2 mg WAT were weighed up in 2 mL reaction tubes, suspended in MeOH (LCMS grade, 500 μL) and 10 μL ISTD Mix (10 μm palmitic acid‐d_31_, stearic acid‐d_35_, oleic acid‐d_9_, arachidonic acid‐d_8_ in MeOH, all Cayman Chemical), homogenized with a TissueRuptor II (Qiagen) and precipitated overnight at −80°C. Supernatants were transferred into 2 mL reaction tubes, concentrated *in vacuo* (RVC 2–25 CD Plus, Martin Christ GmbH, Settings: 1450 rpm, 50°C, 3 mbar, 1 h) and the residue was suspended in ACN (170 μL, Merck). External Standards (10 nM, 30 nM, 0.1 μM, 0.3 μM, 1 μM, 3 μM, 10 μM and 30 μM) in ACN (160 μL) + 10 μL ISTD Mix were used as calibration and controls. Dansyl hydrazine (Merck, 30 μL, 75 mM in ACN, 2.25 μmol, 1.50 equiv.), HATU (Merck, 51 mM in ACN, 1.00 equiv.) and triethylamine (Merck, 30 μL, 3 mM in ACN) were added, and samples were vortexed at 4°C for 2 h. Formic acid (3 μL, 79.5 μmol, 1.56 equiv.) was added, and the solution was allowed to warm to r.t. over 30 min. Samples were concentrated *in vacuo*, the residue was dissolved in ACN (300 μL) and transferred into 300 μL LCMS sample vials. Samples were stored at (−80°C) prior to measurement.

### 2.10 LCMS Measurements

Chromatographic separation was performed on an LCMS‐8050 triple quadrupole mass spectrometer (Shimadzu Duisburg, Germany) interfaced with a Dual Ion Source and a Nexera X3 Front‐End‐System (Shimadzu Duisburg, Germany). Chromatographic separation for S1P and ceramides was performed with a 2 × 60 mm MultoHigh‐C18 RP column with 3 μM particle size at 40°C. MS settings for S1P measurements were the following: interface: ESI, nebulizing gas flow: 3 L/min, heating gas flow: 10 L/min, interface temperature: 300°C, desolvation temperature: 526°C, DL temperature: 250°C, heat block temperature: 400°C, drying gas flow: 10 L/min. MS settings for ceramides were the following: interface: APCI, nebulizing gas flow: 2.4 L/min, heating gas flow: 3 L/min, interface temperature: 300°C, desolvation temperature: 526°C, DL temperature: 250°C, heat block temperature: 400°C, drying gas flow: 3 L/min. Flow rate was 0.4 mL/min. Mobile phases consisted of [A] = MeOH and [B] = aq. HCO_2_H (1% *v/v*) and the following gradient settings were used: [A] increased from 10% to 100% over 3 min (B.curve = −2) and returned to 10% from 8.01 to 10 min prior to the next injection. Data were collected using multiple reaction monitoring (MRM) and positive ionization was used for qualitative analysis and quantification. Standard curves were generated by measuring increased amounts of analytes (100 fmol to 50 pmol C_17_‐S1P, Cer d18:1/14:0, d18:1/16:0, d18:1/18:0, d18:1/18:1, d18:1/20:0, d18:1/22:0, d18:1/24:0, d18:1/24:1 and d17:1/18:0) with an internal standard (S1P d:18:1‐d_7_ = 1 pmol, Cer d18:1/15:0 = 3 pmol). Injection volume of all samples was 10 μL and the following MRM transitions (positive mode) were used for quantification: *m*/*z* = 366.2 → 250.1, 348.2, 268.3 for C_17_‐S1P, *m*/*z* = 387.2 → 271.25 for S1P‐d_7_, [M + H]^+^ → 264 for Cer d18:1/X:Y, and [M + H]^+^ → 250 for Cer d17:1/X:Y. Linearity of standard curves and correlation coefficients were obtained by linear regression analysis. Fatty acid measurements were performed with a 2 × 60 mm MultoHigh‐C8 RP column with 3 μM particle size at 40°C and were detected as their corresponding dansylhydrazides. Mobile phases consisted of [A] = ACN + 0.01% HCO_2_H (v/v) and [B] = 5 mM aq. NH_4_(HCOO) + 0.01% (v/v) HCO_2_H, and the following gradient settings were used: [A] = 40% to 95% from 0 to 9.5 min (B.Curve = −2), 95% to 100% from 9.5 to 12 min, hold to 13 min, and return to 40% from 13.1 to 15 min prior to the next injection. Injection volume of all samples was 3 μL and the MRM transitions [M + H]^+^ → 171 were used for quantification. Linearity of standard curves and correlation coefficients were obtained by linear regression analysis. All MS analyses were performed with LabSolutions 5.114, analyzed with LabSolutions Insight (Shimadzu, Kyoto, Japan) and further processed in Microsoft Excel.

### 2.11 *In Vitro* Kinase Assays With Recombinant or Immunoprecipitated PKC Zeta

For in vitro kinase assays using recombinant enzyme, 150 ng recombinant human PKC zeta protein no. ab60848 (Abcam) was incubated with 5 μg recombinant dephosphorylated MBP (Merck, #13‐110) and 10 μM S1P or methanol as vehicle control in reaction buffer containing 140 μM phosphatidylserine, 2 μM ATP, 10 μCi [32P]‐ATP (Hartmann Analytic), 2.5 mM Tris/HCl pH 7.5, 5 μM EGTA, 50 μM DTT, and 3.75 mM Mg(CH3COO)2 at 30°C for 30 min. The reaction was stopped by adding Laemmli sample buffer (BioRad) before subjecting samples to SDS‐PAGE. Coomassie staining of gels was performed followed by autoradiography. For in vitro kinase assays using immunoprecipitated PKC zeta, gWAT (stored at −80°C; 250 mg) was homogenized in M‐PER Mammalian Protein Extraction Reagent (2 mL) + Halt Protease‐ und Phosphatase‐Inhibitor‐Cocktail (both Thermo Scientific) and incubated on ice for 30 min. Samples were centrifuged (16 000 × *g*, 15 min) and the supernatant was transferred into 2 mL reagent tubes. Protein concentrations were determined using a BCA protein assay (Thermo Scientific). Immunoprecipitation was performed with 750 μg protein and 10 μg PKC zeta (H‐1) agarose‐conjugated (AC) antibody (sc‐17 781 AC, Santa Cruz Biotechnology) overnight at 4°C. Beads were washed in M‐PER Mammalian Protein Extraction Reagent (2 mL, Thermo Scientific) + Halt Protease‐ und Phosphatase‐Inhibitor‐Cocktail and centrifuged (2000 × *g*, 1 min, both Thermo Scientific) 3 times. Kinase assays were performed exactly as above. All experiments are performed as biological replicates.

### 2.12 Statistical Analysis

The statistical significance of differences between groups was evaluated using paired or non‐paired two‐tailed *t*‐tests or a one‐way ANOVA with Tukey's multiple‐comparisons test (GraphPad Prism 5.0; GraphPad Software, La Jolla, CA, USA) as indicated in the figure legends. Data were tested for normality and equal variance before analysis. All results are expressed as mean ± SEM.

## 3 Results

### 3.1 Activation of PKC Zeta by S1P Stimulates Lipolysis in Differentiated 3T3‐L1 Adipocytes

**FIGURE 1:**
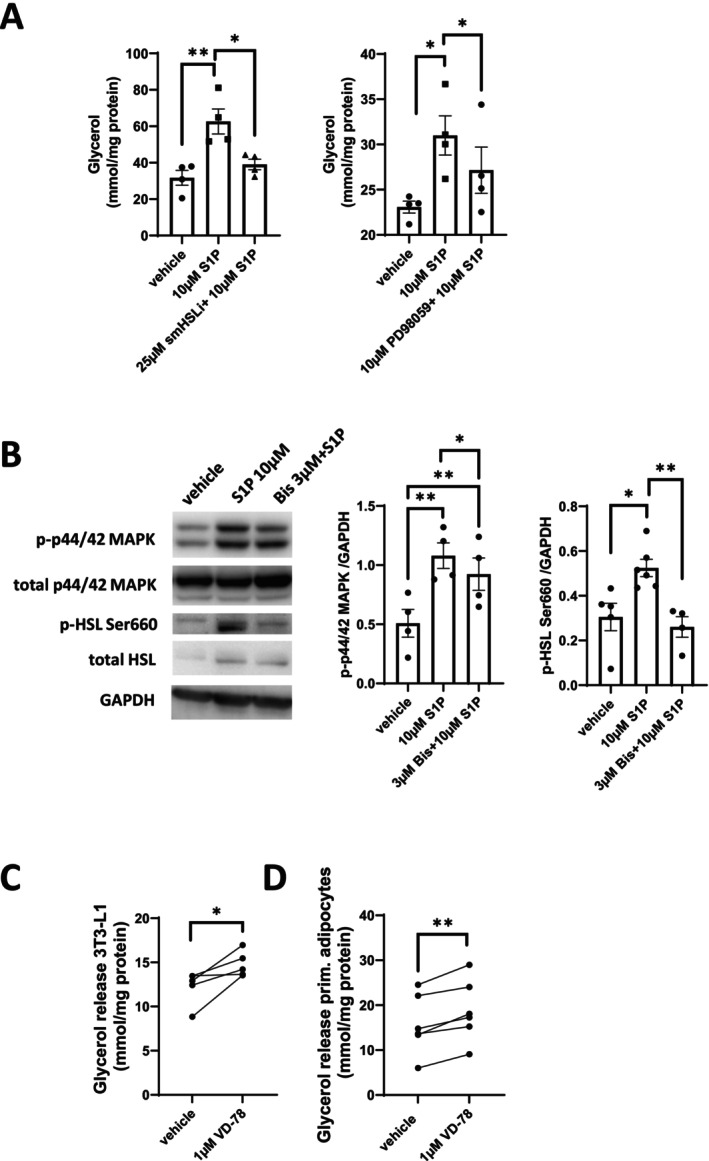
S1P induces lipolysis through PKCzeta in differentiated 3T3‐L1 adipocytes. (A) Glycerol release per mg protein after 16 h treatment of differentiated 3T3‐L1 cells with S1P (0.1, 1, 10 or 50 μM) or vehicle controls (0.1% MeOH or 0.02% BSA) (*n* = 4 per group). (B) Glycerol release per mg protein after 16 h treatment of differentiated 3T3‐L1 cells with 10 μM S1P, 10 μM AUY954, 10 μM CYM5520, 10 μM CYM5541, or vehicle control (0.1% DMSO). (*n* = 4–5 per group). (C) Adipocyte‐associated S1P, sphingosine, and ceramide in 3T3‐L1 adipocytes after a 30 min incubation with 10 μM S1P as measured by LC–MS/MS and calculated based on an average cell volume of 4380 μm^3^. (*n* = 5 per group). (D) Glycerol release per mg protein after 16 h treatment of differentiated 3T3‐L1 cells with 10 μM S1P or vehicle control (0.02% BSA) in the presence or absence of the 25 μM ceramide synthase inhibitor Fumonisin B1. (*n* = 5 per group). *p < 0.05; **p < 0.01.

To investigate whether S1P induces lipolysis, 3T3‐L1 pre‐adipocytes were differentiated into mature adipocytes for 3 weeks, treated with 0.1, 1, and 10 μM S1P for 16 h, and glycerol release into the supernatant was measured to assess lipolysis. We observed that S1P concentrations usually sufficient to activate S1PRs (0.1–1 μM) were ineffective, whereas 10–50 μM S1P increased lipolysis by twofold and threefold, respectively (Figure 1A). Furthermore, stimulation with the S1PR agonists AUY954, CYM5520, and CYM5541 had no effects on lipolysis (Figure 1B). This suggested that S1PR‐independent intracellular mechanisms were involved in S1P‐mediated lipolysis. Indeed, S1P supplemented to the media efficiently and rapidly entered 3T3‐L1 cells in considerable amounts: incubation with 10 μM S1P for 30 min resulted in a 19.3‐fold increase corresponding to 0.17 μM cell‐associated S1P as measured by LCMS and calculated based on adipocyte cell volume (Figure 1C). Sphingosine increased only 5.86‐fold, and ceramides (Cer 14:0, 16:0, 18:0, 22:0, 24:0, and 24:1) did not change at all (Figure 1C). Neither was S1P‐mediated lipolysis affected by the ceramide synthase inhibitor Fumonisin B1 (Figure 1D).

S1P has been reported to activate lipolysis in rat white adipocytes in a cAMP‐dependent manner [14] but we observed no inhibition of S1P‐mediated lipolysis by the adenylate cyclase inhibitor SQ22536, whereas it completely inhibited isoproterenol‐induced lipolysis (Figure 1E). Inhibition of phosphatidylinositol 3‐kinase (PI3K) had no effect on lipolysis (Figure 1F). In search of intracellular S1P targets that may possibly affect lipolysis, we came upon a study showing S1P to bind to the kinase domain and allosterically activate protein kinase C (PKC) zeta [18], an atypical PKC that has been implicated in PMA‐induced lipolysis in mature 3T3‐L1 adipocytes independently of PKA [19]. Using in vitro kinase activity assays with recombinant PKC zeta, we observed that 10 μM S1P clearly stimulated enzyme activity (Figure 1G). We then tested whether inhibiting PKC zeta would have an effect on S1P‐induced lipolysis. Indeed, both the general PKC inhibitor bisindolylmaleinid I (Bis) and a PKC zeta‐specific pseudo‐substrate inhibitor (PSI) completely inhibited S1P‐induced lipolysis (Figure 1H,I). Furthermore, S1P stimulation induced Thr410 phosphorylation in the PKC zeta activation loop required for a catalytically competent enzyme [20] (Figure 1J).

### 3.2 S1P Induces Lipolysis Through a PKC Zeta/MAPK/HSL Pathway

**FIGURE 2:**
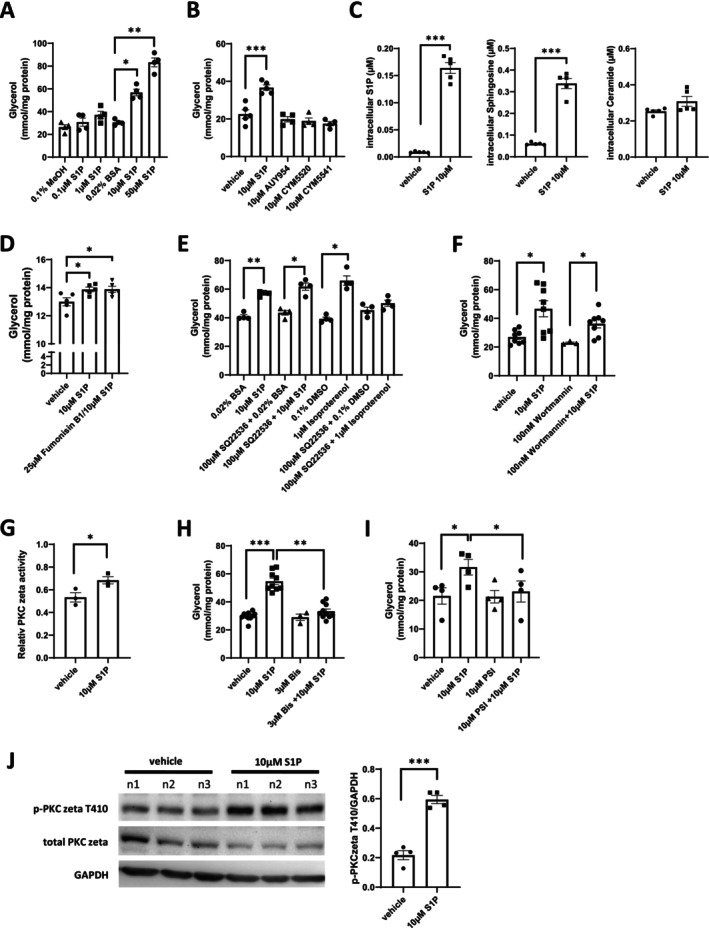
S1P induces lipolysis through a PKCzeta/MAPK/HSL pathway in differentiated 3T3‐L1 cells. (A) Glycerol release per mg protein after 16 h treatment of differentiated 3T3‐L1 cells with vehicle (0.02% BSA), 10 μM S1P (in 0.02% BSA) in the presence or absence of 10 μM PD98059 or 25 μM small molecular HSL inhibitor (smHSLi). (*n* = 4 per group). (B) Representative Western blots for p‐p44/42 MAPK and p‐HSL Ser660 in differentiated T3‐L1 adipocytes treated with 10 μM S1P or vehicle (0.02% BSA) in the presence or absence of 3 μM bisindolylmaleimid I (Bis) for 15 min (*n* = 4–5 per condition). Quantification of p‐p44/42 MAPK and p‐HSL Ser660 normalized to GAPDH. (C) Glycerol release per mg protein after 16 h treatment of differentiated 3T3‐L1 cells with vehicle or 1 μM S1P lyase inhibitor VD‐78. (*n* = 5 per group). (D) Glycerol release per mg protein after 16 h treatment of primary mouse adipocytes with vehicle or 1 μM S1P lyase inhibitor VD‐78 (*n* = 6 per group). Data are presented as mean ± SEM. A one‐way ANOVA with Tukey's multiple‐comparisons test was used for statistical analysis. (E) Gylcerol release per mg protein after 16h treatment of differentiated 3T3‐L1 cells with vehicle (0.02% BSA) vs 10 µM S1P or vehicle (0.1% DMSO) versus 1 µM Isoproterenol in the presence or absence of 100 µM of the adenylate cyclase inhibitor SQ22536. (*n* = 4 per group). (F) Glycerol release per mg protein after 16 h treatment of differentiated 3T3‐L1 cells with 10 µM S1P or vehicle control (0.02% BSA) in presence or absence of the 100 nM PI3K Inhibitor Wortmannin. (*n* = 3–8 per group). (G) Relative kinase activity of recombinant PKC zeta treated with 10 µM S1P or vehicle (MeOH) for 10 min as determined by in vitro phosphorylation assay. (*n* = 3 per group). (H) Glycerol release per mg protein after 16 h treatment of differentiated 3T3‐L1 cells with 10 µM S1P or vehicle control (0.02% BSA) in presence or absence of the 3 µM PKC inhibitor bisindolylmaleimid I (Bis). (*n* = 3–8 per group). (I) Glycerol release per mg protein after 16 h treatment of differentiated 3T3‐L1 cells with 10 µM S1P or vehicle (0.02% BSA) in the presence or absence of 10 µM PKC zeta pseudo‐substrate inhibitor (PSI). (*n* = 4 per group). (J) Representative Western blots for T410 phosphorylated PKC zeta, total PKC zeta and GAPDH in differentiated 3T3‐L1 adipocytes treated with 10 µM S1P or vehicle (0.02% BSA) for 15 min. (*n* = 4 per condition). Quantification of phospho‐PKC zeta T410 normalized GAPDH. *p < 0.05; **p < 0.01; ***p < 0.001.

We next examined the signaling pathway downstream of PKC zeta responsible for S1P‐induced lipolysis. Inhibition of adipose hormone‐sensitive lipase (HSL) by small molecule HSL inhibitor (smHSLi) abolished lipolysis (Figure 2A). In agreement with mitogen‐activated protein kinases (MAPKs) being able to activate HSL by Ser660 phosphorylation in mature 3T3‐L1 adipocytes [21], inhibition of MAPK activation by PD98059 also inhibited lipolysis (Figure 2A). Most importantly, PKC inhibition with Bis prevented both MAPK and HSL phosphorylation by S1P (Figure 2B). These data suggested that S1P stimulated lipolysis by a PKC zeta/MAPK/HSL pathway.

### 3.3 S1P Lyase Inhibition Induces Lipolysis in vitro and In Vivo

**FIGURE 3:**
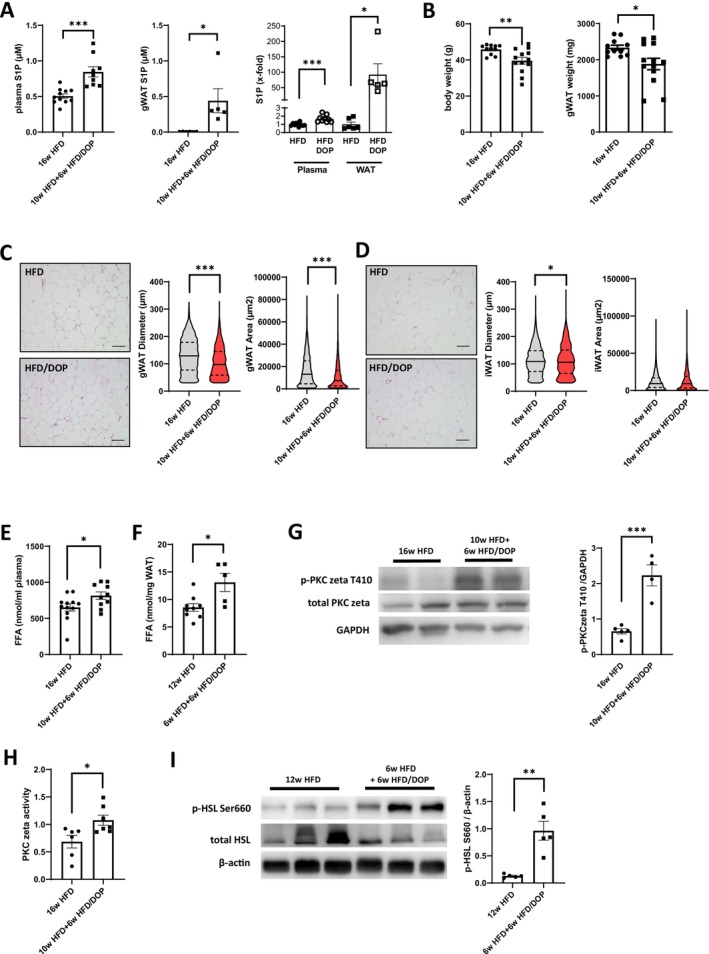
S1P lyase inhibition increases free fatty acids and reduces gWAT mass in HFD‐fed mice due to PKC zeta/HSL activation. (A) Absolute S1P concentrations and relative S1P increase in plasma and gWAT of male C57BL/6J mice after 16 weeks of HFD versus 10 weeks HFD + 6 weeks HFD/DOP treatment. (*n* = 5–11 mice per group). (B) Body weight (left) and gWAT weight (right) of male C57BL/6J mice after 16 weeks of HFD versus 10 weeks HFD + 6 weeks HFD/DOP treatment. (*n* = 11–13 mice per group). (C) Representative H&E staining of perigonadal WAT from male C57BL/6J mice after 16 weeks of HFD versus 10 weeks HFD + 6 weeks HFD/DOP treatment (left). Quantification of adipocyte diameter and area (right). (*n* = 11–13 mice per group). (D) Representative H&E staining of inguinal WAT (iWAT) from male C57BL/6J mice after 16 weeks of HFD versus 10 weeks HFD + 6 weeks HFD/DOP treatment (left) and quantification of adipocyte diameter and area (right). (*n* = 4–5 mice per group). (E) Plasma free fatty acids (FFA) of male C57BL/6J mice after 16 weeks of HFD versus 10 weeks HFD + 6 weeks HFD/DOP treatment. (*n* = 10–12 mice per group). (F) FFA in gWAT of male C57BL/6J mice after 12 weeks of HFD versus 6 weeks HFD + 6 weeks HFD/DOP treatment. (*n* = 5–11 mice per group). (G) Representative Western blots of p‐PKC zeta T410 in gWAT of male C57BL/6J mice after 16 weeks of HFD versus 10 weeks HFD + 6 weeks HFD/DOP treatment. Quantification of p‐PKC zeta T410 to GAPDH. (*n* = 4–5 mice per group). (H) PKC zeta kinase activity as measured by in vitro phosphorylation assay with immunoprecipitated PKC zeta in gWAT of male C57BL/6J mice after 16 weeks of HFD versus 10 weeks HFD + 6 weeks HFD/DOP treatment. (*n* = 6–7 mice per group). (I) Representative Western blots of p‐HSL Ser660 in gWAT of male C57BL/6J mice after 12 weeks of HFD versus 6 weeks HFD + 6 weeks HFD/DOP treatment. Quantification of p‐HSL Ser660 to total beta‐actin. (*n* = 5 mice per group). Data are presented as mean ± SEM. A two‐tailed *t*‐test was used for statistical analysis. *p < 0.05; **p < 0.01; ***p < 0.001.

To test whether not only an increase of extracellular but also intracellular S1P resulted in the induction of lipolysis, we treated differentiated 3T3‐L1 adipocytes with the S1P lyase inhibitor VD‐78 for 16 h and measured glycerol release into the supernatant to assess lipolysis. S1P lyase inhibition resulted in a 21% higher lipolysis in 3T3‐L1 cells (Figure 2C), which was also true for differentiated primary adipocytes (Figure 2D). We then investigated whether increasing S1P levels in vivo had any effect on lipolysis in mice already rendered obese by a high‐fat diet (HFD). To test this, mice were fed a HFD for 10 weeks and then treated with the S1P lyase inhibitor 4‐Deoxypyridoxine (DOP) for an additional 6 weeks of HFD. After the 16 weeks, DOP treatment resulted in a ~2‐fold increase in S1P in plasma and a staggering 92‐fold higher S1P concentration in gonadal white adipose tissue (gWAT) compared to a 16‐week HFD alone control group (Figure 3A). This coincided with a 14% decrease in body weight and a 19% decrease in gWAT mass compared to HFD mice (Figure 3B). Histomorphometric analysis of gWAT and inguinal adipose tissue (iWAT) demonstrated lower adipocyte size (mean diameter and area) and a prominent shift toward smaller cells in DOP‐treated mice compared to mice on HFD alone (Figure 3C,D).

To test whether S1P lyase inhibition and reduced WAT mass coincided with increased lipolysis, we measured free fatty acids (FFA) in plasma and WAT. Indeed, FFA were 25% higher in plasma and 100% higher in WAT, respectively, of DOP‐treated HFD mice as compared to HFD controls (Figure 3E,F). We also measured PKC zeta T410 phosphorylation as well as PKC zeta activity in WAT and observed both to be ~2‐fold higher in DOP‐treated HFD compared to untreated HFD mice (Figure 3G,H). Finally, we detected ~2‐fold higher HSL Ser660 phosphorylation levels in DOP‐treated HFD mice compared to HFD controls (Figure 3I). These results suggested that high S1P levels due to S1P lyase inhibition induced lipolysis in vivo by the same mechanism as in vitro, resulting in reduced adiposity.

## 4 Discussion

This study is the first to assign a physiological function to the activation of PKC zeta by S1P, namely lipolysis. We suggest this to occur through intracellular S1P activating PKC zeta that then turns on MAPK/ERK signaling resulting in HSL activation. This argues in favor of intracellular effects as S1P was efficient only at concentrations between 5 and 50 μM (that exceed by far the Kd of S1PRs) but not at lower concentrations (0.1–1 μM), and because the S1PR1–3 receptor agonists AUY954, CYM5520, and CYM5541 were ineffective. In addition, while several S1PRs are known to activate PI3K signaling that in turn can activate PKC zeta [22, 23], PI3K inhibition did not abolish S1P's lipolytic effect in our study. Using LCMS, we could also trace the fate of extracellularly added S1P and observed it to accumulate in considerable (μM) concentration in adipocytes, whereas ceramide levels did not increase. Furthermore, increasing endogenous intracellular S1P by inhibition of the S1P lyase using VD‐78 resulted in increased lipolysis in 3T3‐L1 cells and primary adipocytes. Our data on higher lipolysis in vivo after raising whole body S1P by S1P lyase inhibition support our findings in vitro including those on PKC zeta and HSL activation by S1P, and demonstrate their physiological consequences in the form of decreased adipocyte size, adipose tissue mass, and body weight.

PKC zeta belongs to the two atypical PKC isoforms (zeta and lambda in mice/iota in humans) that are not activated by diacylglycerols (DAG) [24] but through binding to protein scaffolds and lipid mediators, respectively [25]. They are co‐translationally phosphorylated by mTORC2, followed by post‐translational phosphorylation by PDK1 at the activation loop, resulting in a catalytically‐competent enzyme [26, 27]. Atypical PKCs are maintained in an inactive auto‐inhibited conformation by the interaction of the basic pseudosubstrate region (PSR) [25] and the DAG‐insensitive C1 domain [28] with the kinase domain. Activation occurs, in general, by conformational changes that relieve auto‐inhibition by tethering the PSR and C1 away from the substrate‐binding cavity [25], leading to the phosphorylation of co‐localized substrates to increase efficiency considering their 40‐fold lower catalytic rate compared to conventional PKCs (cPKC) [27]. A unique feature of aPKCs is their activation not only by binding to protein scaffolds but also through interactions with lipids such as phosphatidic acid (PA), phosphatidylserine (PS), possibly phosphatidylinositol 3,4,5‐trisphosphate (PI [3, 4, 5]) and by sphingolipids such as ceramides and S1P [29]. Ceramides were shown to activate PKC zeta [30, 31] through binding to amino acids 405–592 in the carboxyl‐terminal 20‐kDa sequence [32] and to activate kinase activity at sub‐nanomolar concentrations (maximum at 3–10 nM) [33]. In contrast, S1P binds to a pocket on the surface of the kinase domain, thereby reducing auto‐inhibitory contacts with the pseudosubstrate‐C1 module [18]. Treatment with 10 μM S1P did not alter ceramide levels in our experiments, nor did inhibition of ceramide synthase affect S1P‐induced lipolysis, suggesting that ceramides did not play a role.

In a metabolic context, PKC zeta activation by PI3K contributes to insulin‐stimulated glucose uptake in white and brown adipocytes [22, 23]. However, excessive aPKC activity in diabetes and obesity contributes to insulin resistance, for example, in HFD‐fed mice, overtly diabetic or insulin‐resistant ob/ob mice, diabetic rats, and humans. Excessive aPKC activation can be caused by increased ceramide levels, and its consequence is de‐sensitization of Akt signaling. This occurs by displacement of Akt from the WD40/ProF platform, thus preventing Akt‐mediated FoxO1 phosphorylation [34, 35] and Thr34 phosphorylation in the Akt‐PH domain preventing PIP3 binding [36], respectively. In the context of lipogenesis, PI3K‐activated aPKCs are instrumental for insulin‐dependent increases in SREBP‐1c expression and lipid uptake [37]. Accordingly, interventions designed to decrease ceramide accumulation, such as overexpression of acid ceramidase in liver or adipose tissue, have been shown to reverse insulin resistance and hepatic steatosis by inhibiting PKC zeta and preventing its insulin‐desensitizing and lipid uptake/fatty acid synthesis‐promoting effects [38].

Currently, the known stimulatory effects of ceramides on lipolysis have not been put in relation to aPKC but rather to their interference with cAMP/PKA signaling, as ceramides have been shown to suppress ß‐adrenoreceptor‐induced HSL activation and lipolysis by activating Protein‐Phosphatase 2 (PP2A) in brown adipocytes [39]. Interestingly, PMA‐induced activation of aPKCs has been implicated in activating lipolysis in differentiated 3T3‐L1 adipocytes by activating HSL through MAPK/ERK, a pathway that acted independently of and synergistically with cAMP/PKA‐dependent lipolysis in the same cells [19]. Other stimuli such as lipopolysaccharide (LPS) and Gi‐coupled G protein‐coupled receptor agonists such as lysophosphatidic acid (LPA) have also been shown to induce MEK/MAPK/ERK activation by activating PKC zeta through ceramide [40] or in a RAS‐independent manner [41]. Our data support a cAMP/PKA‐independent MAPK/ERK‐dependent lipolytic effect of PKC zeta. They also provide S1P as the first intracellular physiological activator in this pathway. The inability of PI3K inhibition to abolish the S1P effect suggests that S1P short‐circuits any possible PI3K‐mediated PKC zeta activation. We must acknowledge that our data rely on inhibitor studies alone, but all our attempts to introduce for example, dominant‐negative PKC zeta using retroviral infection were unsuccessful in the notoriously difficult‐to‐transduce 3T3‐L1 cells [42].

Studies on S1P effects explicitly on obesity or adipocyte biology in vivo are complicated by their many associated co‐morbidities [1, 43], whereas studies in humans mostly measure S1P in the circulation and are difficult to interpret as they range from S1P positively correlating with body mass index (BMI) [44] to others showing curvilinear associations [4]. Understanding the role of S1P specifically in adipocyte physiology and pathophysiology will help decipher one of the important aspects of obesity and obesity‐associated disorders.

## Author Contributions

**Sarah Weske:** investigation, methodology, visualization, formal analysis, writing – original draft preparation. **Melissa Kim Nowak:** investigation, methodology, visualization, formal analysis, writing – original draft preparation. **Alex Zaufel:** investigation, formal analysis, methodology. **Lea Esser:** investigation, formal analysis, methodology. **Christoph Peter:** investigation, formal analysis, methodology. **Helena Kühn:** investigation, formal analysis, methodology. **Lisa Walz:** investigation, formal analysis, methodology. **Tsyon Wolde:** investigation, formal analysis, methodology. **Julia Hoppe:** investigation, formal analysis, methodology. **Nathalie Hannelore Schröder:** investigation, formal analysis, methodology. **Tobias Buschmann:** investigation, methodology, formal analysis, writing – reviewing and editing. **Philipp Wollnitzke:** investigation, methodology, formal analysis, writing – reviewing and editing. **Bodo Levkau:** conceptualization, investigation, validation, data interpretation, writing – original draft preparation, writing – reviewing and editing, supervision, project administration, funding acquisition.

## Conflicts of Interest

The authors declare no conflicts of interest.

## Data Availability

All data are contained within the manuscript.

## References

[1] K. Kajita , I. Ishii , I. Mori , M. Asano , M. Fuwa , and H. Morita , “Sphingosine 1‐Phosphate Regulates Obesity and Glucose Homeostasis,” International Journal of Molecular Sciences 25, no. 2 (2024): 932. 38256005 10.3390/ijms25020932 PMC10816022

[2] S. Ali‐Berrada , J. Guitton , S. Tan‐Chen , A. Gyulkhandanyan , E. Hajduch , and H. Le Stunff , “Circulating Sphingolipids and Glucose Homeostasis: An Update,” International Journal of Molecular Sciences 24, no. 16 (2023): 12720. 37628901 10.3390/ijms241612720 PMC10454113

[3] B. Ramos‐Molina , J. Rossell , A. Perez‐Montes de Oca , et al., “Therapeutic Implications for Sphingolipid Metabolism in Metabolic Dysfunction‐Associated Steatohepatitis,” Frontiers in Endocrinology 15 (2024): 1400961. 38962680 10.3389/fendo.2024.1400961 PMC11220194

[4] T. Hashimoto , J. Igarashi , and H. Kosaka , “Sphingosine Kinase Is Induced in Mouse 3T3‐L1 Cells and Promotes Adipogenesis,” Journal of Lipid Research 50 (2009): 602–610. 19020339 10.1194/jlr.M800206-JLR200 PMC2656653

[5] M. H. Moon , J. K. Jeong , Y. J. Lee , J. W. Seol , and S. Y. Park , “Sphingosine‐1‐Phosphate Inhibits the Adipogenic Differentiation of 3T3‐L1 Preadipocytes,” International Journal of Molecular Medicine 34 (2014): 1153–1158. 25050633 10.3892/ijmm.2014.1856

[6] M. H. Moon , J. K. Jeong , and S. Y. Park , “Activation of S1P2 Receptor, a Possible Mechanism of Inhibition of Adipogenic Differentiation by Sphingosine 1‐Phosphate,” Molecular Medicine Reports 11 (2015): 1031–1036. 25351259 10.3892/mmr.2014.2810

[7] J. K. Jeong , M. H. Moon , and S. Y. Park , “Modulation of the Expression of Sphingosine 1‐Phosphate 2 Receptors Regulates the Differentiation of Pre‐Adipocytes,” Molecular Medicine Reports 12 (2015): 7496–7502. 26459774 10.3892/mmr.2015.4388

[8] M. H. Moon , J. K. Jeong , J. H. Lee , et al., “Antiobesity Activity of a Sphingosine 1‐Phosphate Analogue FTY720 Observed in Adipocytes and Obese Mouse Model,” Experimental & Molecular Medicine 44 (2012): 603–614. 22859500 10.3858/emm.2012.44.10.069 PMC3490082

[9] S. E. Kim , J. H. Lee , M. K. Chae , E. J. Lee , and J. S. Yoon , “The Role of Sphingosine‐1‐Phosphate in Adipogenesis of Graves' Orbitopathy,” Investigative Ophthalmology & Visual Science 57 (2016): 301–311. 26830367 10.1167/iovs.15-17863

[10] S. Weske , M. Vaidya , A. Reese , et al., “Targeting Sphingosine‐1‐Phosphate Lyase as an Anabolic Therapy for Bone Loss,” Nature Medicine 24 (2018): 667–678. 10.1038/s41591-018-0005-y 29662200

[11] S. Weske , M. Vaidya , K. von Wnuck Lipinski , et al., “Agonist‐Induced Activation of the S1P Receptor 2 Constitutes a Novel Osteoanabolic Therapy for the Treatment of Osteoporosis in Mice,” Bone 125 (2019): 1–7. 31028959 10.1016/j.bone.2019.04.015

[12] Y. Kitada , K. Kajita , K. Taguchi , et al., “Blockade of Sphingosine 1‐Phosphate Receptor 2 Signaling Attenuates High‐Fat Diet‐Induced Adipocyte Hypertrophy and Systemic Glucose Intolerance in Mice,” Endocrinology 157 (2016): 1839–1851. 26943364 10.1210/en.2015-1768 PMC4870879

[13] X. Wu , M. K. Sakharkar , M. Wabitsch , and J. Yang , “Effects of Sphingosine‐1‐Phosphate on Cell Viability, Differentiation, and Gene Expression of Adipocytes,” International Journal of Molecular Sciences 21, no. 23 (2020): 9284. 33291440 10.3390/ijms21239284 PMC7730007

[14] D. J. Jun , J. H. Lee , B. H. Choi , et al., “Sphingosine‐1‐Phosphate Modulates Both Lipolysis and Leptin Production in Differentiated Rat White Adipocytes,” Endocrinology 147 (2006): 5835–5844. 16973728 10.1210/en.2006-0579

[15] A. K. Anderson , J. M. Lambert , D. J. Montefusco , et al., “Depletion of Adipocyte Sphingosine Kinase 1 Leads to Cell Hypertrophy, Impaired Lipolysis, and Nonalcoholic Fatty Liver Disease,” Journal of Lipid Research 61 (2020): 1328–1340. 32690594 10.1194/jlr.RA120000875 PMC7529052

[16] C. Kilkenny , W. J. Browne , I. C. Cuthill , M. Emerson , and D. G. Altman , “Improving Bioscience Research Reporting: The ARRIVE Guidelines for Reporting Animal Research,” Journal of Pharmacology and Pharmacotherapeutics 1 (2010): 94–99. 21350617 10.4103/0976-500X.72351 PMC3043335

[17] J. Oeckl , A. Bast‐Habersbrunner , T. Fromme , M. Klingenspor , and Y. Li , “Isolation, Culture, and Functional Analysis of Murine Thermogenic Adipocytes,” STAR Protocols 1 (2020): 100118. 33377014 10.1016/j.xpro.2020.100118 PMC7757011

[18] T. Kajimoto , A. D. Caliman , I. S. Tobias , et al., “Activation of Atypical Protein Kinase C by Sphingosine 1‐Phosphate Revealed by an aPKC‐Specific Activity Reporter,” Science Signaling 12, no. 562 (2019): eaat6662, 10.1126/scisignal.aat6662. 30600259 PMC6657501

[19] K. Fricke , A. Heitland , and E. Maronde , “Cooperative Activation of Lipolysis by Protein Kinase A and Protein Kinase C Pathways in 3T3‐L1 Adipocytes,” Endocrinology 145 (2004): 4940–4947. 15284193 10.1210/en.2004-0803

[20] I. S. Tobias , M. Kaulich , P. K. Kim , et al., “Protein Kinase Czeta Exhibits Constitutive Phosphorylation and Phosphatidylinositol‐3,4,5‐Triphosphate‐Independent Regulation,” Biochemical Journal 473 (2016): 509–523. 26635352 10.1042/BJ20151013 PMC4888060

[21] A. S. Greenberg , W. J. Shen , K. Muliro , et al., “Stimulation of Lipolysis and Hormone‐Sensitive Lipase via the Extracellular Signal‐Regulated Kinase Pathway,” Journal of Biological Chemistry 276 (2001): 45456–45461. 11581251 10.1074/jbc.M104436200

[22] G. Bandyopadhyay , M. P. Sajan , Y. Kanoh , et al., “PKC‐Zeta Mediates Insulin Effects on Glucose Transport in Cultured Preadipocyte‐Derived Human Adipocytes,” Journal of Clinical Endocrinology and Metabolism 87 (2002): 716–723. 11836310 10.1210/jcem.87.2.8252

[23] M. Arribas , A. M. Valverde , D. Burks , et al., “Essential Role of Protein Kinase C Zeta in the Impairment of Insulin‐Induced Glucose Transport in IRS‐2‐Deficient Brown Adipocytes,” FEBS Letters 536 (2003): 161–166. 12586357 10.1016/s0014-5793(03)00049-8

[24] A. C. Newton , “Protein Kinase C as a Tumor Suppressor,” Seminars in Cancer Biology 48 (2018): 18–26. 28476658 10.1016/j.semcancer.2017.04.017 PMC5668200

[25] M. L. Drummond and K. E. Prehoda , “Molecular Control of Atypical Protein Kinase C: Tipping the Balance Between Self‐Renewal and Differentiation,” Journal of Molecular Biology 428 (2016): 1455–1464. 26992354 10.1016/j.jmb.2016.03.003 PMC4848065

[26] J. A. Le Good , W. H. Ziegler , D. B. Parekh , D. R. Alessi , P. Cohen , and P. J. Parker , “Protein Kinase C Isotypes Controlled by Phosphoinositide 3‐Kinase Through the Protein Kinase PDK1,” Science 281 (1998): 2042–2045. 9748166 10.1126/science.281.5385.2042

[27] I. S. Tobias , M. Kaulich , P. K. Kim , et al., “Protein Kinase Cζ Exhibits Constitutive Phosphorylation and Phosphatidylinositol‐3,4,5‐Triphosphate‐Independent Regulation,” Biochemical Journal 473 (2016): 509–523. 26635352 10.1042/BJ20151013 PMC4888060

[28] H. Zhang , S. Neimanis , L. A. Lopez‐Garcia , et al., “Molecular Mechanism of Regulation of the Atypical Protein Kinase C by N‐Terminal Domains and an Allosteric Small Compound,” Chemistry & Biology 21, no. 6 (2014): 754–765, 10.1016/j.chembiol.2014.04.007. 24836908

[29] S. Velnati , S. Centonze , F. Girivetto , et al., “Identification of Key Phospholipids That Bind and Activate Atypical PKCs,” Biomedicine 9 (2021): 45. 10.3390/biomedicines9010045 PMC7825596 33419210

[30] N. A. Bourbon , J. Yun , and M. Kester , “Ceramide Directly Activates Protein Kinase C Zeta to Regulate a Stress‐Activated Protein Kinase Signaling Complex,” Journal of Biological Chemistry 275 (2000): 35617–35623. 10962008 10.1074/jbc.M007346200

[31] T. E. Fox , K. L. Houck , S. M. O'Neill , et al., “Ceramide Recruits and Activates Protein Kinase C Zeta (PKC Zeta) Within Structured Membrane Microdomains,” Journal of Biological Chemistry 282, no. 17 (2007): 12450–12457, 10.1074/jbc.M700082200. 17308302

[32] G. Wang , K. Krishnamurthy , N. S. Umapathy , A. D. Verin , and E. Bieberich , “The Carboxyl‐Terminal Domain of Atypical Protein Kinase Czeta Binds to Ceramide and Regulates Junction Formation in Epithelial Cells,” Journal of Biological Chemistry 284 (2009): 14469–14475. 19304661 10.1074/jbc.M808909200 PMC2682895

[33] G. Müller , M. Ayoub , P. Storz , J. Rennecke , D. Fabbro , and K. Pfizenmaier , “PKC Zeta Is a Molecular Switch in Signal Transduction of TNF‐Alpha, Bifunctionally Regulated by Ceramide and Arachidonic Acid,” EMBO Journal 14 (1995): 1961–1969. 7744003 10.1002/j.1460-2075.1995.tb07188.x PMC398295

[34] M. P. Sajan , R. A. Ivey , M. C. Lee , and R. V. Farese , “Hepatic Insulin Resistance in Ob/Ob Mice Involves Increases in Ceramide, aPKC Activity, and Selective Impairment of Akt‐Dependent FoxO1 Phosphorylation,” Journal of Lipid Research 56 (2015): 70–80. 25395359 10.1194/jlr.M052977 PMC4274073

[35] M. P. Sajan , M. E. Acevedo‐Duncan , M. L. Standaert , R. A. Ivey , M. Lee , and R. V. Farese , “Akt‐Dependent Phosphorylation of Hepatic FoxO1 Is Compartmentalized on a WD40/ProF Scaffold and Is Selectively Inhibited by aPKC in Early Phases of Diet‐Induced Obesity,” Diabetes 63 (2014): 2690–2701. 24705403 10.2337/db13-1863 PMC4113067

[36] D. J. Powell , E. Hajduch , G. Kular , and H. S. Hundal , “Ceramide Disables 3‐Phosphoinositide Binding to the Pleckstrin Homology Domain of Protein Kinase B (PKB)/Akt by a PKCzeta‐Dependent Mechanism,” Molecular and Cellular Biology 23 (2003): 7794–7808. 14560023 10.1128/MCB.23.21.7794-7808.2003 PMC207567

[37] C. M. Taniguchi , T. Kondo , M. Sajan , et al., “Divergent Regulation of Hepatic Glucose and Lipid Metabolism by Phosphoinositide 3‐Kinase via Akt and PKClambda/Zeta,” Cell Metabolism 3 (2006): 343–353. 16679292 10.1016/j.cmet.2006.04.005

[38] J. Y. Xia , W. L. Holland , C. M. Kusminski , et al., “Targeted Induction of Ceramide Degradation Leads to Improved Systemic Metabolism and Reduced Hepatic Steatosis,” Cell Metabolism 22 (2015): 266–278. 26190650 10.1016/j.cmet.2015.06.007 PMC4527941

[39] B. Chaurasia , L. Ying , C. L. Talbot , et al., “Ceramides Are Necessary and Sufficient for Diet‐Induced Impairment of Thermogenic Adipocytes,” Molecular Metabolism 45 (2021): 101145. 33352310 10.1016/j.molmet.2020.101145 PMC7807150

[40] M. M. Monick , A. B. Carter , D. M. Flaherty , M. W. Peterson , and G. W. Hunninghake , “Protein Kinase C Zeta Plays a Central Role in Activation of the p42/44 Mitogen‐Activated Protein Kinase by Endotoxin in Alveolar Macrophages,” Journal of Immunology 165 (2000): 4632–4639. 10.4049/jimmunol.165.8.4632 11035106

[41] H. Takeda , T. Matozaki , T. Takada , et al., “PI 3‐Kinase Gamma and Protein Kinase C‐Zeta Mediate RAS‐Independent Activation of MAP Kinase by a Gi Protein‐Coupled Receptor,” EMBO Journal 18 (1999): 386–395. 9889195 10.1093/emboj/18.2.386 PMC1171133

[42] D. J. Orlicky , J. DeGregori , and J. Schaack , “Construction of Stable Coxsackievirus and Adenovirus Receptor‐Expressing 3T3‐L1 Cells,” Journal of Lipid Research 42 (2001): 910–915. 11369798

[43] P. J. Larsen and N. Tennagels , “On Ceramides, Other Sphingolipids and Impaired Glucose Homeostasis,” Molecular Metabolism 3 (2014): 252–260. 24749054 10.1016/j.molmet.2014.01.011 PMC3986510

[44] G. M. Kowalski , A. L. Carey , A. Selathurai , B. A. Kingwell , and C. R. Bruce , “Plasma Sphingosine‐1‐Phosphate Is Elevated in Obesity,” PLoS One 8 (2013): e72449. 24039766 10.1371/journal.pone.0072449 PMC3765451

